# Proteome Profiling by Label‐Free Mass Spectrometry Reveals Differentiated Response of *Campylobacter jejuni* 81–176 to Sublethal Concentrations of Bile Acids

**DOI:** 10.1002/prca.201800083

**Published:** 2018-10-11

**Authors:** Wycliffe O. Masanta, Andreas E. Zautner, Raimond Lugert, Wolfgang Bohne, Uwe Gross, Andreas Leha, Mohammed Dakna, Christof Lenz

**Affiliations:** ^1^ Institute for Medical Microbiology University Medical Center Göttingen 37075 Göttingen Germany; ^2^ Department of Medical Microbiology Maseno University Medical School Private Bag 40105 Maseno Kenya; ^3^ Department of Medical Statistics University Medical Center Göttingen 37073 Göttingen Germany; ^4^ Bioanalytical Mass Spectrometry Group Max Planck Institute for Biophysical Chemistry 37077 Göttingen Germany; ^5^ Institute of Clinical Chemistry Bioanalytics University Medical Center Göttingen 37075 Göttingen Germany

**Keywords:** bile acids, *Campylobacter jejuni*, mass spectrometry, proteome, SWATH

## Abstract

**Purpose:**

Bile acids are crucial components of the intestinal antimicrobial defense and represent a significant stress factor for enteric pathogens. Adaptation processes of *Campylobacter jejuni* to this hostile environment are analyzed in this study by a proteomic approach.

**Experimental design:**

Proteome profiling by label‐free mass spectrometry (SWATH‐MS) has been used to characterize the adaptation of *C. jejuni* to sublethal concentrations of seven bile acids.

**Results:**

The bile acids with the lowest inhibitory concentration (IC_50_), deoxycholic and chenodeoxycholic acid, induce the most significant proteome changes. Overall a downregulation of all basic biosynthetic pathways and a general decrease in the transcription machinery are found. Concurrently, an induction of factors involved in detoxification of reactive oxygen species, protein folding, and bile acid exporting efflux pumps is detected. Exposure to deoxycholic and chenodeoxycholic acid results in an increased expression of components of the more energy‐efficient aerobic respiration pathway, while the anaerobic branches of the electron transport chain are down‐expressed.

**Conclusions and clinical relevance:**

The results show that *C. jejuni* has a differentiated system of adaptation to bile acid stresses. The findings enhance the understanding of the pathogenesis of campylobacteriosis, especially for survival of *C. jejuni* in the human intestine, and may provide clues to future medical treatment.

## 1 Introduction

*Campylobacter jejuni* is the leading cause of bacterial gastroenteritis worldwide. Symptoms include diarrhea, fever, and stomach cramps.^1^ Several weeks after acute campylobacteriosis post‐infectious sequelae like Guillain‐Barré syndrome may follow.^2^ In the human gut, *C. jejuni* resides mainly in the jejunum.^3^, ^4^ Bile acids are one of the major constituents of intestinal fluid and inhibit microbial growth.^5^, ^6^ They are grouped based on their origin: primary bile acids are synthesized in the liver comprising (among others) cholic acid (CA), chenodeoxycholic acid (CDCA), taurocholic acid (TCA), glycocholic (GCA), glycochenodeoxycholic (GCDCA), and taurochenodeoxycholic acid (TCDCA),^7^, ^8^ while the group of secondary bile acids is produced in the colon and comprises deoxycholic acid (DCA) and, lithocholic acid (LCA). Tertiary bile acids like ursodeoxycholic acid (UDCA) are bile acids that are reconjugated in the liver after passing the enterohepathic circle.^9^ Accordingly, intestinal pathogens such as *C. jejuni* are confronted with these bile acids that are primarily toxic to bacteria after ingestion.^10^ However, little is known about the adaptation of *C. jejuni* to the resulting stress under physiological conditions.

Label‐free quantitative mass spectrometry represents the state of the art for comprehensive proteome profiling of microbial systems under environmental stresses.^11^ The experimental approach involves cell harvest and lysis, isolation and endopeptidase digestion of proteins, separation of peptides by liquid chromatography (LC), and identification and quantification from mass spectrometry data (MS and/or MS/MS) by parallelized acquisition.^12^ One of the most powerful implementations of label‐free quantitative mass spectrometry is SWATH‐MS (sequential window acquisition of all theoretical mass spectra).^13^, ^14^ SWATH‐MS employs an ion library typically derived from data‐dependent acquisition (DDA) to extract chromatographic peak areas from data‐independent acquisition (DIA) where fragments of all precursors in the sample have been recorded using sequential *m/z* precursor windows.^15^, ^16^ The technique has been found to generate accurate quantification results comparable to, e.g., isotope labeling‐based techniques such as iTRAQ.^17^, ^18^ It routinely allows for single‐shot, low‐bias expression profiling of full proteome samples to a depth of 2000–4000 proteins, which is a perfect match for the complexity of most microbial proteomes. SWATH‐MS has consequently been used to investigate clinically relevant systems, such as in vivo host–pathogen interactions of *Staphylococcus aureus* 19 or the dormancy cycle of *Mycobacterium tuberculosis*.^20^

In this study we have used SWATH‐MS to investigate the stress response of *C. jejuni* strain 81–176 to sublethal concentrations of CA, CDCA, TCA, GCA, DCA, LCA, and UDCA.

## 2 Experimental Section

### 2.1 Determination of Bile Acid Half Maximal Inhibitory Concentrations (IC_50_) and Evaluation of Growth at Half IC_50_ Concentrations for *Campylobacter jejuni* 81–176

Bile acids were purchased from SIGMA–Aldrich (Taufkirchen, Germany): CA (C9282), CDCA (C8261), DCA (D2510), LCA (L6250), TCA (T4009), UDCA (U5127), and GCA (G7132).

A dilution series was prepared for each bile acid using *Campylobacter* defined broth (CDB),^21^ a minimum medium containing only the amino acids and vitamins essential for *Campylobacte*r growth, containing 1.5, 0.75, 0.375, 0.1875, 0.09375, 0.046875, 0.0234375, 0.01171875, and 0.0% m/m (control = pure CDB; Ctr) of each bile acid, respectively.

**Clinical Relevance**

Infection by *Campylobacter jejuni* is the most common cause of bacterial gastroenteritis worldwide. Here, it must be taken into account that not only acute enteritis but especially post‐infectious diseases such as reactive arthritis and the Guillain‐Barré syndrome (GBS) are a high cost burden for healthcare systems.

Bile acids play an essential role in intestinal antimicrobial defense. They are a major component of the chyme passing the small intestines where bacterial pathogens like *C. jejuni* thrive. Little is known about how *C. jejuni* adapts to the stress of specific bile acids in the human small intestines. However, it must be anticipated that in the context of the stress response to bile acids, the proteome will change significantly and specific proteins will be up‐expressed while others will be down‐expressed.

The identification of the cellular processes that enable *C. jejuni* to adapt to sublethal concentrations of major primary, secondary bile, and tertiary bile acids will significantly improve the understanding of the pathogenesis of acute campylobacteriosis. Proteins that are up‐expressed in the bile acid stress response may be specific epitopes of the anti‐*Campylobacter* immune response and thus explain the etiology of post‐infectious complications and may be used as antigens in new serological test panels.

*Campylobacter jejuni* 81–176 stored in cryo stocks was cultured on Columbia agar base (Merck) supplemented with 5% sheep blood (BA) and incubated at 42 °C under microaerobic (6.2–13.2% ≈ 10% O_2_) and carbon dioxide enriched (2.5–9.5% ≈ 5% CO_2_) conditions using the CampyGen gas generating system (ThermoScientific, Hampshire, UK). Bacterial colonies grown on solid agar were transferred to liquid medium by means of an inoculation loop and suspended by pipetting up and down.

The OD_600_ of *C. jejuni* 81–176 growing in CDB for 16 h at 37 °C under microaerophilic conditions while shaking (Edmund Bühler GmbH, Bodelshausen, Germany) at 150 rpm was measured. This culture was adjusted to an OD_600_ of 0.1. Subsequently, 1.5 mL of each bile acid dilution (double concentrated with regard to final dilution) and 1.5 mL of *C. jejuni* 81–176 suspension (resulting in an OD_600_ of 0.05) were mixed and the 3.0 mL suspension was further incubated for 16 h at 37 °C. After incubation the OD_600_ of each suspension was recorded. Each experiment was performed in duplicate at three different occasions. Finally, a graph of OD_600_ versus concentration (mm) of *C. jejuni*’s growth in response to each bile acid was drawn and the intercept section of the two bile acid concentrations directly flanking the obvious IC_50_ value was taken to calculate the precise IC_50_ value of each bile acid. Growth curves of *C. jejuni* 81–176 in CDB supplemented with 50% of the respective IC_50_ were determined for each bile acid to ensure inhibitory but sublethal effects (Figure S1, Supporting Information). Again, each measurement was performed in duplicate at three different occasions.

### 2.2 MS Sample Preparation

*Campylobacter jejuni* 81–176 was cultured in 3.0 mL CDB (37 °C for 12 h, shaken at 150 rpm, under microaerobic (6.2–13.2% ≈ 10%O_2_) and carbon dioxide enriched (2.5–9.5% ≈ 5%CO_2_) conditions) supplemented with the following bile acid concentrations: [CA] = 0.075% (1.74 mm), [CDCA] = 0.05% (1.21 mm), [TCA] = 0.485% (9.02 mm), [GCA] = 0.370% (7.59 mm), [DCA] = 0.030% (0.72 mm), [LCA] = 0.500% (13.28 mm), and [UDCA] = 0.485% (12.35 mm). As a control, a culture with pure CDB was used. The time of harvest after 12 was chosen because here the growth curves for the different bile acids are close to each other (Figure S1, Supporting Information) and thus competing effects due to, e.g., stress caused by a lack of nutrients in consequence of excessive bacterial cell density can be largely excluded. Similarly, bile acid concentrations of ½ IC_50_ were chosen to largely exclude a non‐specific stress response due to high levels of toxin, and possibly even proteomic changes due to cell death.

Cultures were harvested by centrifuging at 1700 × *g* for 10 min. Pellets were resuspended in 1 mL of 0.9% aqueous sodium chloride solution. The suspensions were sonicated on ice using five bursts at a setting of 3 and 30% duty cycles (Branson Model 250) for 30 s with 30 s intervals. Cell debris was removed by centrifuging at 15 300 × *g* at 4 °C for 15 min. The harvested proteins were separated by 4–12% gradient SDS‐PAGE and visualized by Colloidal Coomassie staining. Protein concentration in the supernatants was quantified performing the Bradford method (λ = 595 nm). Finally, the extracted proteins were purified by acetone precipitation (acetone:sample 4:1, v/v,  –20 °C, overnight). Protein pellets were washed with ice‐cold acetone, air‐dried, and redissolved using sodium 3‐[(2‐methyl‐2‐undecyl‐1,3‐dioxolan‐4‐yl)‐methoxy]‐1‐propanesulfonate (cleavable surfactant, Rapigest, Waters, Eschborn, Germany). After reduction and alkylation of cysteine residues with dithiothreitol and iodoacetamide, proteins were digested using sequencing grade porcine trypsin (Promega, Mannheim, Germany) at a 1:50 enzyme‐to‐substrate ratio (w:w). Following acidic cleavage of the surfactant, the resulting fatty acids were pelleted and removed by centrifugation. The resulting peptide mixtures were dried in a SpeedVac Concentrator centrifuge (Thermo Scientific, Darmstadt, Germany) and stored at −20 °C prior to analysis. Three biological replicates were prepared for each bile acid and for the controls (pure CDB) and then analyzed.

### 2.3 LC–MS/MS Acquisition

Protein digests were analyzed on a Nanoflow chromatography system (Eksigent nanoLC425, SCIEX, Darmstadt, Germany) hyphenated to a hybrid triple quadrupole‐time of flight mass spectrometer (TripleTOF 5600+, SCIEX, Darmstadt, Germany) equipped with a Nanospray III ion source (Ionspray Voltage 2200 V, Interface Heater Temperature 150 °C, Sheath Gas Setting 10) and controlled by Analyst TF 1.6 software (all AB Sciex, Darmstadt, Germany). In brief, peptides from each digest were dissolved in loading buffer (2% aqueous acetonitrile vs 0.1% formic acid) to a concentration of 0.5 μg μL^–1^, desalted on a trap column (Dr. Maisch RP‐C18aq, particle size 5 μm, 30 × 0.150 mm, 60 μL loading buffer) and separated by reversed phase‐C18 nanoflow chromatography (Dr. Maisch, Ammerbuch‐Entringen, Germany; RP‐C18aq, particle size 3 μm, 250 × 0.075 mm, linear gradient 90 min 5% > 35% acetonitrile versus 0.1% formic acid, 300 nL min^–1^, 50 °C).

Qualitative LC–MS/MS analysis was performed using a Top25 data‐dependent acquisition (DDA) method with an MS survey scan of *m/z* 380–1250 accumulated for 250 ms at a resolution of 35 000 FWHM (full width at half maximum). MS/MS scans of *m/z* 180–1750 were accumulated for 100 ms at a resolution of 17 500 FWHM and a precursor isolation width of 0.7 FWHM, resulting in a total cycle time of 3.4 s. Precursors above a threshold MS intensity of 200 cps with charge states 2+, 3+, and 4+ were selected for MS/MS, the dynamic exclusion time was set to 15 s. Two technical replicates of 1.5 μg protein equivalent of one biological sample per condition were acquired for qualitative analysis, and combined for protein identification and generation of a spectral library for targeted data extraction.

For data‐independent acquisition (DIA) by SWATH analysis, MS/MS data were acquired for 100 precursor segments of variable size (5–40 Th) resulting in a precursor *m/z* range of 400–1250. Fragments were produced using rolling collision energy settings and fragments acquired over an *m/z* range of 380–1600 for an accumulation time of 40 ms per segment. Including a 250 ms survey scan this resulted in an overall cycle time of 4.5 s. Three technical replicates of 2.0 μg protein equivalent of three biological replicates per condition (3 × 3 replication scheme) were acquired for quantitative analysis.

### 2.4 LC–MS/MS Data Processing

Protein identification was achieved using ProteinPilot V5.0 (AB Sciex, Darmstadt, Germany) at “thorough” settings. A total of 551.443 MS/MS spectra from the combined qualitative analyses were searched against the *C. jejuni* 81–176 proteome from UniProtKB (1804 protein entries) supplemented with 51 commonly observed lab and workflow contaminants. Global false discovery rates (FDR) were adjusted to 1% at both the protein and peptide level using a forward/reverse decoy database approach.

SWATH peak extraction was achieved in PeakView V2.1 (AB Sciex, Darmstadt, Germany) using the SWATH quantitation microApp V2.0. Following retention time alignment on a set of 12 endogenous peptides, peak areas were extracted for up to the eight highest scoring peptides per protein group at six transitions per peptide, an extracted ion current (XIC) width of 75 ppm and an XIC window of 8 min, and filtered to an estimated FDR of 1%.

The mass spectrometry data have been deposited to the ProteomeXchange Consortium via the PRIDE^22^ partner repository with the dataset identifier PXD009088.

### 2.5 Statistical and Bioinformatics Analysis

The resulting peak areas were exported at the fragment, peptide and protein level for further statistical analysis. The Empirical Bayes method for Mixed Models implemented in the R Bioconductor limma package^23^ was used to determine proteins that were significantly up‐ or down‐expressed in 81–176 by each bile acid.^24^ Before analysis, UniProt accessions were substituted by gene names. Subsequently, mixed model analysis was performed in two stages: in the first stage, regression coefficients of the influence of each bile acid on the expression of each gene were determined independently. In the second stage, the regression coefficients of each bile acid were compared in a single equation to create a relationship on influence of expression on genes between the bile acids. Finally, moderated *t*‐statistics were used to measure protein expression changes between different bile acid stimuli. Because the same linear model is fitted to each protein allows us to borrow strength between proteins in order to moderate the residual variances. The estimated variance for each protein then becomes a compromise between the protein‐wise estimator, obtained from the data for that protein alone, and the global variability across all proteins, estimated by pooling the ensemble of all proteins. This has the effect of increasing the effective degrees of freedom with which the protein‐wise variances are estimated.

Proteins that showed at least a 1.5‐fold change in either direction (i.e. log_2_(FC) ≥ 0.585 or log_2_(FC) ≤ ‐0.585) and an FDR‐adjusted *p*‐value less than 0.05 were considered to be significantly differentially expressed.

## 3 Results and Discussion

### 3.1 CA, DCA, LCA, TCA, CDCA, UDCA, and GCA have Different IC_50_ Concentrations

IC_50_ for each of the bile acids were determined (Table S1, Supporting Information). At 1.45, 2.41, and 3.48 mm, respectively, DCA, CDCA, and CA showed the lowest IC_50_, which renders them most toxic to *C. jejuni*. Since DCA, CDCA, and CA form the largest proportions of bile acids in the human intestine at about 20, 35, and 35%,^25^, ^26^ respectively, we will focus on these bile acids for further discussion.

Sublethal concentrations were defined as 50% of the determined IC_50_. These are similar to physiological bile acid concentrations of 0.2 to 2% in the human small intestine^10^ and were subsequently used for proteomic analysis.

### 3.2 Protein Identification by Nano‐LC–MS/MS

Aliquots of each digested protein sample were analyzed by DDA mass spectrometry to identify expressed and detectable proteins, and generate a spectral library for quantitative global proteome profiling by SWATH‐MS. Liu and coworkers had previously reported a proteome coverage of up to 86% in *C. jejuni* 81–176 employing SDS‐PAGE separation followed by tryptic digestion and bottom‐up mass spectrometric analysis. Our single‐shot analysis yielded a set of 1079 proteins substantiated by 14 644 peptide sequences at 1% FDR, respectively. Of these, 1063 were *C. jejuni* proteins, corresponding to identification of 58.9% of the predicted proteome, which is comparable to previous analyses of other bacteria and archaea using the same approach (Table S2, Supporting Information).^27^ Of note is the high peptide‐to‐protein ratio of 13.6, which is a prerequisite for reliable protein quantitation by peptide‐based approaches.

### 3.3 Global Proteome Profiling by SWATH Mass Spectrometry

For global proteome profiling the samples were analyzed by DIA using SWATH mass spectrometry. With the help of a spectral library generated from the peptide‐to‐spectrum matches (PSMs) obtained for protein identification and FDR estimation on the transition level, 957 proteins (or 53.0% of the predicted proteome, Supporting Information Dataset 1) substantiated by 4298 peptides and 25 785 precursors were found to be quantifiable across all replicates (Table S3, Supporting Information).

**Figure 1:**
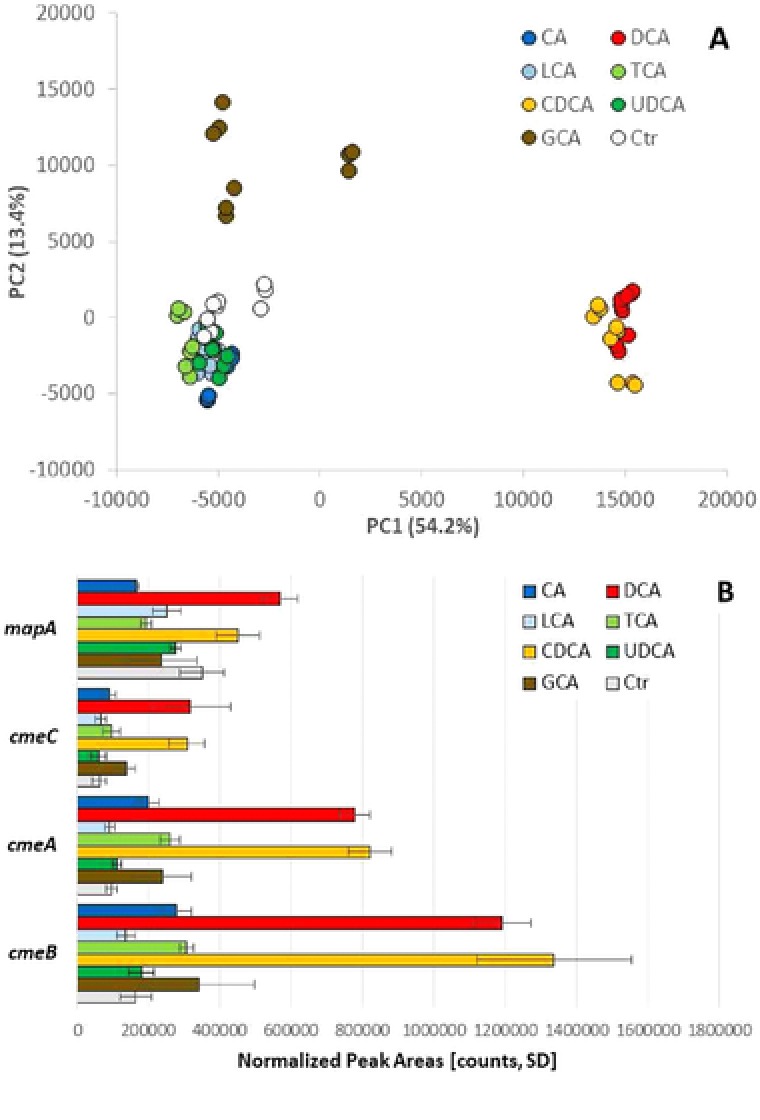
A) PCA analysis displaying the correlation between 3 × 3 biological and technical replicates of *C. jejuni* 81–176 cultured at sublethal concentrations of different bile acids and CDB without bile acids (Ctr, Control) for 12 h at 37 °C. B) Selected SWATH‐MS quantitation results for the toxin MapA and three RND efflux system components CmeABC. Standard deviations represent 3 × 3 biological and technical replicates, respectively.

A non‐directed principal component analysis (PCA) was performed to test for major differences between the different bile acid stimuli, as well as to assess reproducibility among biological and technical replicates (Figure 1A). PCA analysis revealed three major distinguishable sample groups.

The first group comprised CA, LCA, TCA, and UDCA, which did not show a marked difference to the control sample. In a second group, DCA and CDCA stimulated samples clustered distinctly by PC1; and finally GCA stimulated samples were strongly differentiated in PC2, indicating a distinct pattern of protein expression and hinting at a different set of stress responses. Overall the biological differences clearly exceeded the differences between both biological and technical replicates, respectively, indicating good reproducibility of the analytical workflow.

The plausibility of results was further evaluated using the expression levels of known proteins of interest in this context (Figure 1B). For example, previous studies showed that the multidrug efflux transporter CmeABC plays an important role in bile resistance.^28^, ^29^ SWATH‐MS analysis indeed indicated elevated levels of CmeABC proteins across the board, but especially for DCA and CDCA. Interestingly, peak areas anticorrelate with the IC_50_ values for the different bile acids, suggesting that CmeABC expression is a direct measure of the susceptibility of *C. jejuni* to bile acid stress. The expression profile for the surface protein MapA displayed a similar profile, hinting at distinct response patterns to different bile acid stresses.

### 3.4 Identification of Biological Processes Affected by Sublethal Concentrations of Bile Acids

**Figure 2:**
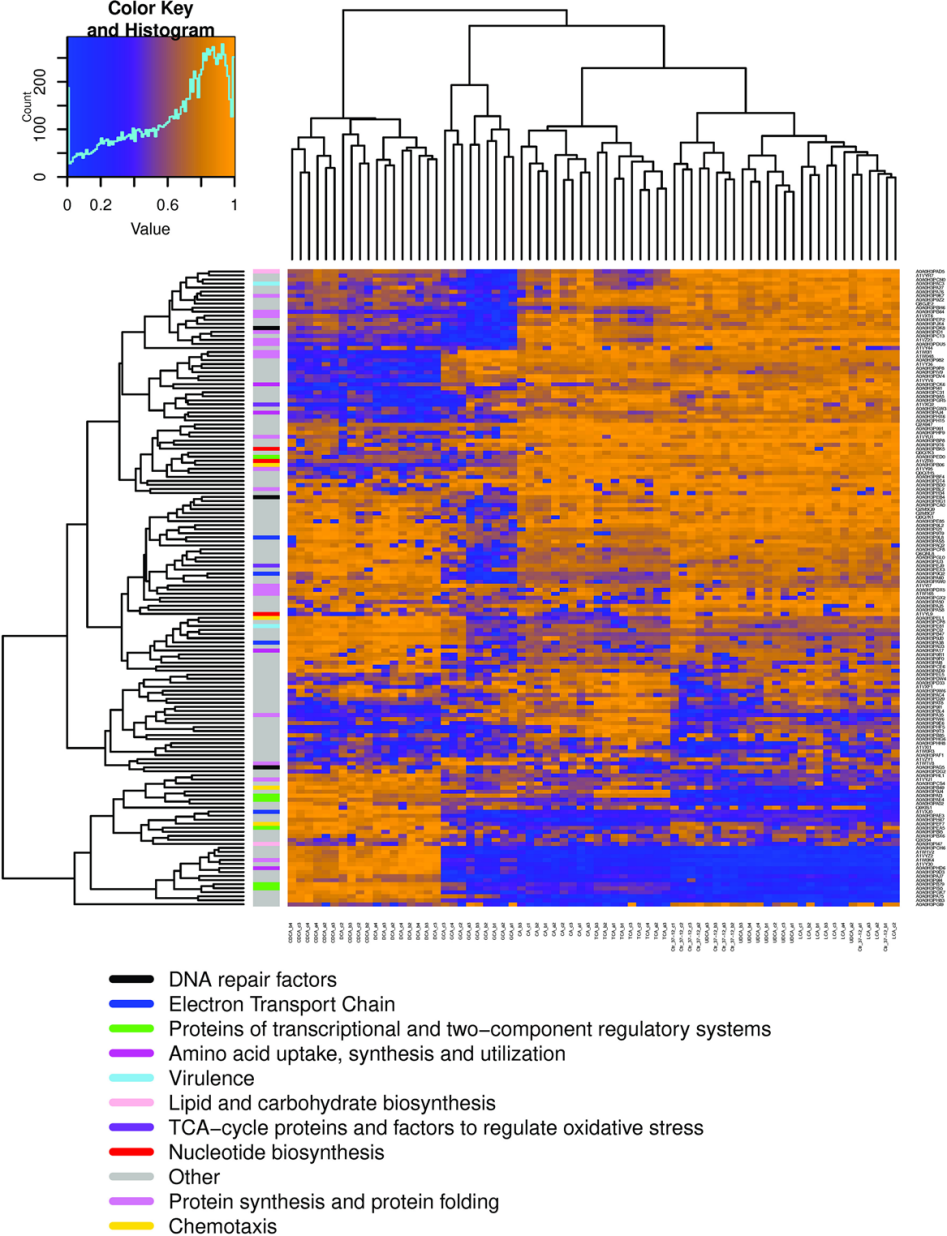
Hierarchical Clustering Analysis of differentially expressed proteins, indicating distinct response patterns for (I) DCA/CDCA, (II) GCA, (III) CA/TCA and (IV) LCA/UDCA stresses; Ctr, Control (CDB without bile acids); color code first column indicating functional groups: black, DNA repair factors; blue, electron transport chain; green, proteins of transcriptional and two‐component regulatory systems; magenta, amino acid uptake, synthesis, and utilization; skyblue, virulence; pink, lipid and carbohydrate biosynthesis; purple, TCA‐cycle proteins and factors to regulate oxidative stress; red, nucleotide biosynthesis; grey, other; violet, protein synthesis and protein folding; yellow, chemotaxis.

Empirical Bayes Analysis for Mixed Models was used to determine proteins that were significantly up‐ or down‐expressed in *C. jejuni* 81–176 under each bile acid stress.^24^ Using this set of 157 differentially expressed proteins, we first performed a Hierarchical Clustering Analysis (HCA) to determine if the bile acid clusters observed in the previous PCA indeed represented corresponding protein expression patterns, which in turn indicate distinct bile acid‐specific stress response patters (Figure 2). This was indeed the case. DCA and CDCA samples showed a distinct protein expression pattern (I) as did GCA samples (II). A third distinct expression pattern for bile acids CA and TCA (III) was clearly different though from the rest of the samples (UDCA, LCA, and CDB (Control); IV), confirming the initial finding that different bile acids trigger distinct stress response patterns on the global protein expression level.

A topical analysis of the available annotations for the corresponding proteins indicated a significant involvement of proteins in, e.g., translation, membrane transport, cell motility, and energy metabolism. We tried to analyze the differentiating protein clusters by enrichment analysis to look for specific biological processes or pathways impacted by the different bile acid sets. The lack of consistent gene ontology or pathway information present for *C. jejuni* 81–176 in the available databases, however, did not permit this data‐driven approach. Instead, we decided to take a biology‐oriented approach and will discuss the observed protein expression changes by biological process against current knowledge. In general, our data show a significant suppression of basic biosynthetic pathways including nucleotide, protein, lipid, and carbohydrate biosynthesis under bile acid stress, as well as a general reduction of the translation machinery (Tables S4–S8, Supporting Information).

*Redox Enzymes and Detoxification of Reactive Oxygen Species (ROS)*: The exact mechanism of how bile acids lead to increased ROS levels in bacteria is incompletely understood. In mitochondria of mammalian cells bile acids were shown to interfere with electron transport chain (ETC) function by alteration the mitochondrial membrane permeability, which finally resulted in increased ROS generation.^30^ Whether a similar mechanism exists in bacteria has not been reported yet. Redox enzymes mediating oxidative phosphorylation through the electron carriers are a main source of ROS in bacteria.^31^ Twelvefold increased ROS levels as a result of exposure of *C. jejuni* to 0.05% DOC have been detected directly by measurement of oxidized 2’,7’‐dichlorodihydrofluorescein diacetate fluorescence.^32^ In bacteria, the bile acid induced ROS lead finally to toxic DNA damage and induce an increase in catalase activity and a decrease in transcripts for redox enzymes that might contribute to ROS production.^32^

From the known proteins that contribute to ROS detoxification,^33^ catalase (KatA) and thiol‐peroxidase (Tpx) were induced in the presence of two and five different bile acids, respectively (Table 1). Down‐expressed enzymes include thioredoxin‐disulfide reductase (TrxB), bacterioferritin comigratory protein (Bcp), and superoxide dismutase (SodB). The up‐expression of catalase KatA and Tpx suggests an adaptation to increased ROS levels. Bingham‐Ramos and coworkers have shown that catalase was the major enzyme involved in H_2_O_2_ detoxification in *C. jejuni*.^34^ In contrast, Atack and Kelly claim that catalase may not be the major enzyme to detoxify hydrogen peroxide under physiological conditions as other enzymes like AhpC, TrxB, and Tpx also take on this task in *C. jejuni*.^33^ Up‐expression of KatA and Tpx at the protein and the transcript levels has also been shown by Rodrigues et al. under aerobic atmosphere.^35^ Furthermore, the thiol peroxidase Tpx, together with Bcp, contributes to the aerotolerance of *C. jejuni*.^36^ Interestingly, a relatively high abundance of catalase and Tpx versus SodB, AhpC and TrxB was also observed during adaptation to intracellular growth.^37^

**Table 1:** TCA‐cycle proteins and factors to regulate oxidative stress

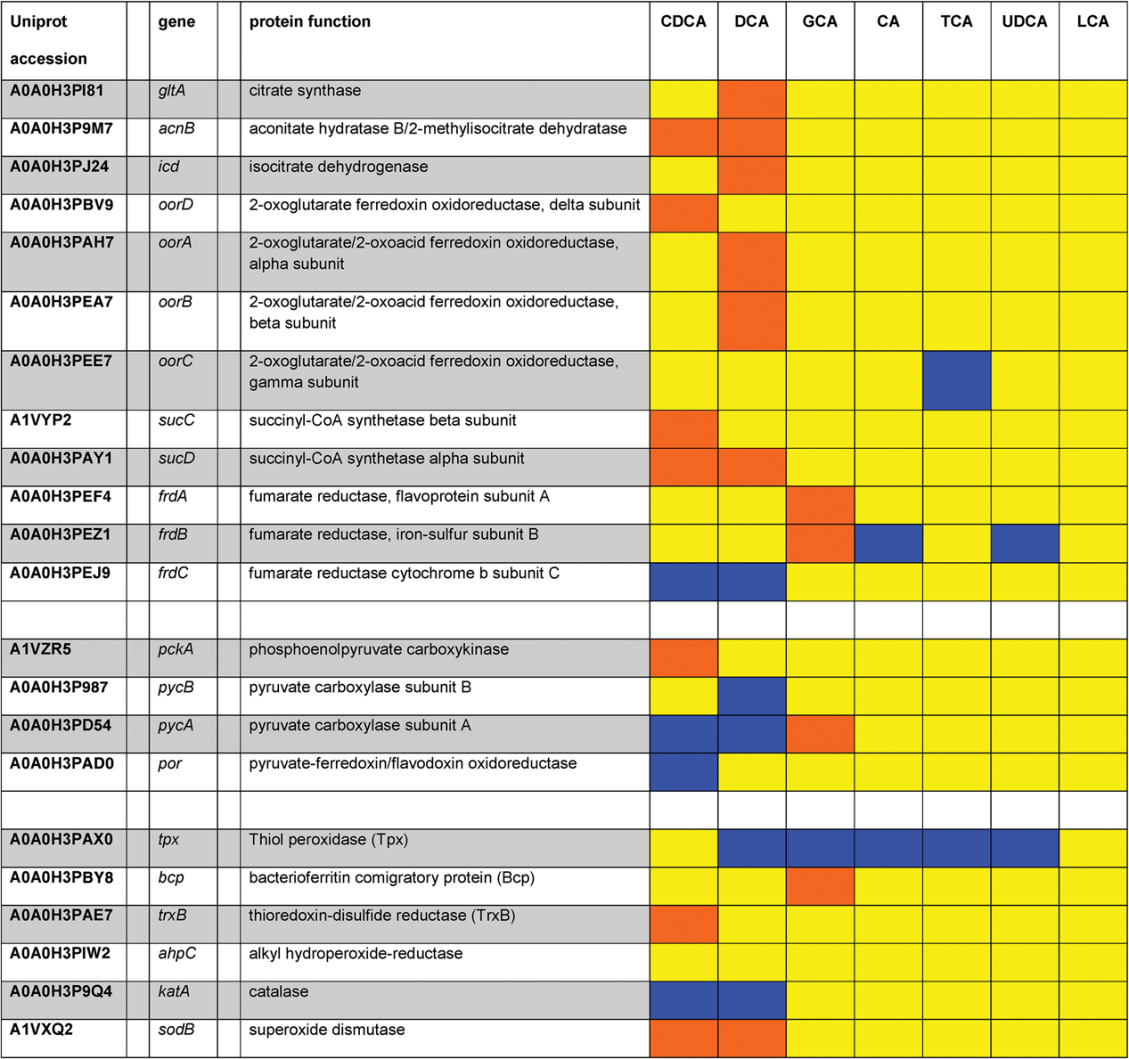

Blue, bile acids with log_2_FC ≥ 0.585 (upregulated); orange, bile acids with log_2_FC ≤ –0.585 (downregulated); yellow, bile acids with ‐0.585 ≤ log_2_FC ≤ 0.585 (not significantly altered in expression); grey, proteins not detected in experimental setting.

ROS generation in bacteria is a side effect of the redox enzyme activity in the ETC when electrons are accidentally transferred to oxygen rather than to the natural substrates of the ETC complexes.^31^ *C. jejuni* is equipped with a highly branched ETC that allows both aerobic and anaerobic metabolism.^38^ A cbb3‐type cytochrome‐c oxidase (complex IV) and a cyanide‐insensitive oxidase both use molecular oxygen as a substrate. In addition, *C. jejuni* possesses a variety of enzymes that can transfer electrons to substrates such as fumarate, nitrate, nitrite, trimethylamine‐*N*‐oxide (TMAO), and dimethylsulfoxide under anaerobic conditions.^37^, ^38^, ^39^

All key components of the aerobic respiration pathway were up‐expressed after exposure to DCA and CDCA. Simultaneously, anaerobic components of the ETC were down‐expressed (Table 2).

**Table 2:** Electron Transport Chain

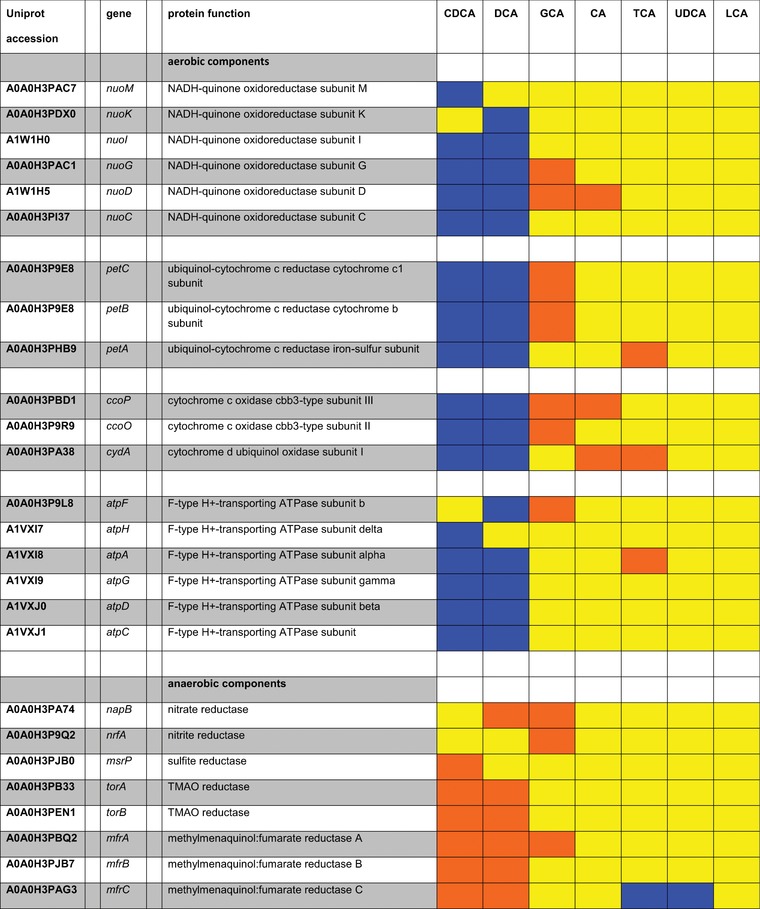

Blue, bile acids with log_2_FC ≥ 0.585 (upregulated); orange, bile acids with log_2_FC ≤ –0.585 (downregulated); yellow, bile acids with ‐0.585 ≤ log_2_FC ≤ 0.585 (not significantly altered in expression); grey, proteins not detected in experimental setting.

The pattern of up‐ and down‐expressed proteins suggests a bile acid induced shift toward oxygen‐dependent respiratory chain activity. Increased ATP synthesis by oxidative phosphorylation might be beneficial and necessary for the pathogen to survive bile acid stress. It remains to be confirmed whether the bile acid‐induced increase in respiratory chain proteins also leads to higher respiratory activity. However, an increased respiratory chain activity would explain the deoxycholate‐induced higher ROS levels observed by Negretti and coworkers.^32^

*Tricarboxylic Acid Cycle*: The effect of bile acids on TCA cycle associated enzymes displays a mixed pattern of up‐ and down‐expressed proteins. The fumarate reductase complex (Frd) is the only enzyme that exhibits succinate dehydrogenase activity in *C. jejuni* and is thus essential for succinate oxidation in the TCA cycle.^40^ This enzyme was up‐expressed in the presence of DCA and CDCA. Other TCA cycle enzymes as citrate synthase, aconitase, isocitrate dehydrogenase, 2‐oxoglutarate oxidoreductase, and succinyl‐CoA synthetase displayed a reduced expression level (Table 1). Enzymes involved in pyruvate metabolism as pyruvate carboxylase, phosphoenolpyruvate carboxykinase, and pyruvate‐ferredoxin oxidoreductase exhibit increased expression levels.

*Transcriptional and Two‐Component Regulatory Systems*: Today, bacterial multidrug efflux pumps are commonly reduced to their role as antimicrobial resistance factors. As it is, these are evolutionary ancestral proteins that are found in all organisms. They can transport a wide range of substrates out of the cell, including heavy metal ions, quorum sensing signals, bacterial metabolites, biocides, organic poisons, and even antiseptics. In particular, resistance to biocidal substances such as bile acids is associated with the virulence of bacterial pathogens.^41^, ^42^, ^43^ One of the first studied and best characterized efflux pumps is the RND (resistance–nodulation–division) transporter AcrAB in *Escherichia coli*, which in addition to various classes of antibiotics also has non‐antibiotic substrates such as dyes, solvents, and detergents (including bile salts).^41^, ^44^

The multidrug efflux pumps in *C. jejuni*, CmeABC, and CmeDEF, consist of a periplasmic fusion protein CmeA/CmeD, an inner membrane efflux transporter CmeB/CmeE and an outer membrane protein CmeC/CmeF. They are known to be involved in bile resistance.^29^ Expression of CmeABC was shown to be regulated by the transcriptional repressor CmeR.^45^ CmeR also regulates the expression of other 28 genes including the periplasmic protein Cj0561c (Cjj81176_0586),^45^ and bile acids were demonstrated to induce the expression of CmeABC and Cj0561c.^46^

A second two‐component regulatory system uses the *Campylobacter* bile resistance regulator (CbrR) to modulate DCA resistance, although its effector mechanisms are not yet known.^47^ Both CmeR and CbrR have been found to play an important role in the colonization of chicken by *C. jejuni*.^45^

Our label‐free proteome analysis confirmed these regulatory mechanisms. Both regulatory repressor proteins CmeR and CbrR were down‐expressed under DCA, CDCA, and GCA exposure. Consequently, all subunits of the CmeABC efflux pump showed increased expression. Furthermore, we could detect elevated expression of the CmeR controlled Cj0561c homologue protein Cjj81176_0586. Regarding the other RND‐efflux pumps encoded in the *C. jejuni* genome, we could detect an increased expression of CmeE.

*Chemotaxis and Flagellar Motility*: Chemotaxis in *C. jejuni* has been studied in great detail and several chemoreceptors and chemoeffectors have been identified,^48^ e.g., CA, DCA, TCA, and GCA have been demonstrated as chemorepellents.^49^

According to our data, different bile acids (Table 3), specifically DCA, CDCA, or GCA, had very different effects. Expression of the three group A chemoreceptors Tlp1, Tlp2, and Tlp10 was increased; in contrast, expression of Tlp4 and group C chemoreceptor Tlp5 was lowered.

**Table 3:** Proteins of transcriptional and two‐component regulatory systems, chemotaxis and virulence associated proteins induced by bile acids

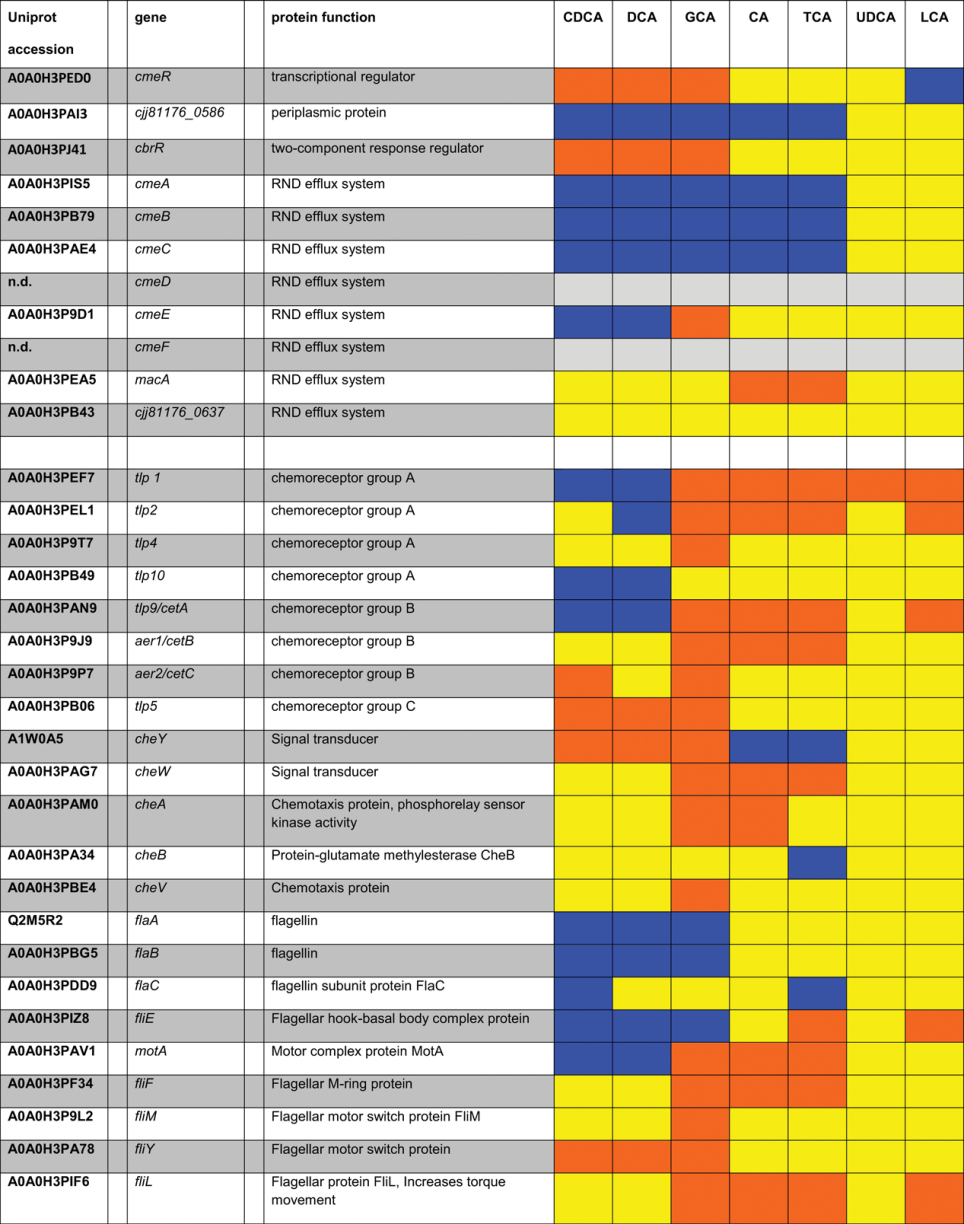
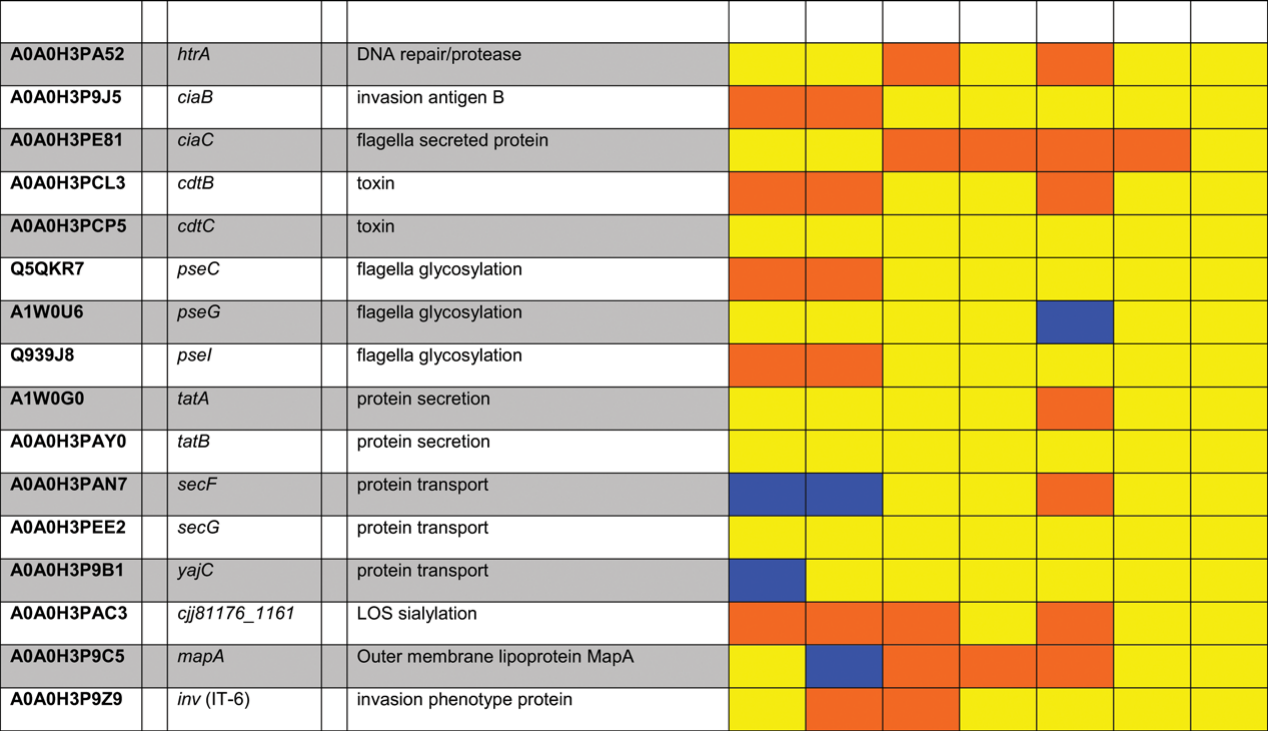

Blue, bile acids with log_2_FC ≥ 0.585 (upregulated); orange, bile acids with log_2_FC ≤ –0.585 (downregulated); yellow, bile acids with ‐0.585 ≤ log_2_FC ≤ 0.585 (not significantly altered in expression); grey, proteins not detected in experimental setting.

The only group B chemoreceptor in *C. jejuni*, Tlp9, sensing oxygen‐related changes in redox potential was increasingly expressed, whereas the two associated aerotaxis proteins Aer1 and Aer2 were less expressed. Additionally four chemoreceptor signal transduction proteins CheW, CheY, CheA, and CheV were less expressed.

Proteins building up external structures of the flagellar apparatus: FlaA/B/C, FliE, and MotA showed an increased expression, whereas the motor/switch proteins of the cytoplasmic C‐ring FliF, FliM, FilY, and FliL were down‐expressed.

Since the external parts of the flagellar apparatus also have other functions than the motility, e.g., epithelial cell adherence, a picture emerges pointing to a specification of the chemotaxis mediated flagellar motility by DCA, CDCA, and GCA and an increase of adhesion factors.

*Virulence‐Associated Factors*: One of the most important *C. jejuni* virulence‐associated proteins is HtrA, a serine protease that acts also as a chaperone. HtrA is able to cleave E‐cadherin and occludin, enabling the bacterium to open tight junctions and access the basolateral compartment. In consequence *C. jejuni* can enter the bloodstream, and reach the mesenteric lymph nodes.^50^ Quantitative mass spectrometry revealed that HtrA expression is downregulated by TCA and GCA (Table 3).

*Campylobacter* invasion antigens (Cia) are proteins that are secreted via the type III‐homologue secretion system of the flagellar apparatus of *C. jejuni*. Eight different Cias have been demonstrated playing a role in the invasion of epithelial cells.^51^ Only two of these, CiaB and CiaC, were significantly changed in their expression by bile acids. Surprisingly, CiaB levels were reduced under DCA and CDCA stress, whereas CiaC levels were reduced by CA, TCA, UDCA, and GCA. Both Cias are delivered to the cytosol and are responsible for host cell cytoskeletal rearrangements.^51^, ^52^ Surprisingly other Cia proteins were not significantly changed in their expression by bile acids due to our data. This is especially remarkable as CiaB has been demonstrated to be induced by DCA by microarray analysis.^53^

The cytolethal distending toxin (CDT) consists of the subunits CdtA, CdtB, and CdtC. It was found to induce cell distension in different mammalian cell lines. The subunits CdtA and CdtC harbor lectin‐like regions and are necessary for binding to the host cell, whereas CdtB is the enzymatically active part of the tripartite holotoxin leading to cell cycle arrest and apoptosis.^1^, ^54^ Our proteomic analysis indicates that the subunit CdtB was reduced in its expression by DCA, TCA, and CDCA, while the expression of the other two subunits is not affected by bile acids.

*O*‐linked glycosylated flagellin is a crucial factor for attachment to intestinal epithelial cells and chicken colonization. It has been shown that defects in *O*‐linked glycosylation result in a loss of motility and in a decrease of adherence to and invasion in host cells.^1^, ^55^ Our study indicates that expression of two proteins involved in pseudaminic acid synthesis, PseC and PSeI, was reduced by DCA and CDCA, while PseG remained unaltered under DCA and CDCA exposure, but it was induced by TCA.

The twin‐arginine translocation (tat) system facilitates the transport of folded proteins across the cytoplasmic membrane that are involved in the adaptation and survival of the bacterial cells, most of them are redox proteins that contribute to the bacterium's branched ETC (see above).^56^ Our data indicate that both, TatA and TatB, remain mostly unaltered in their expression under bile acid influence, only TatA expression was suppressed by TCA.

In bacteria, the sec‐secretion system is the major passageway for protein secretion across the cytoplasmic membrane or insertion of integral membrane proteins into the phospholipid bilayer and it has been shown that high virulent strains have additional enzymes that are sec‐secreted into the periplasmic space.^57^ Two of the proteins facilitating sec‐secretion, SecF and YajC, were induced by DCA or CDCA. SecG expression was not altered by bile acids.

Sialylation of the lipooligosaccharide LOS has been shown to increase epithelial cell invasion.^58^ Due to our data DCA, TCA, CDCA, and GCA suppressed expression of acylneuraminate cytidylyltransferase Cjj81176_1161.

Furthermore, levels of the outer membrane lipoprotein MapA, which was demonstrated to act as fitness factor for chicken colonization,^59^ were increased by DCA, but reduced by CA, TCA, and GCA. DCA and GCA decreased expression of a homologue to the invasion phenotype protein IT‐6.

## 4 Conclusions

We report here the first *C. jejuni* proteome study under influence of different bile acids. A deep look into differential protein expression by label‐free mass spectrometry (SWATH‐MS) revealed that stress response takes place in a highly differentiated manner which can be subdivided into several processes.

Our study demonstrated a significant downregulation of basic biosynthetic pathways, e.g., nucleotide‐, protein‐, lipid‐, and carbohydrate‐biosynthesis, in addition to a general reduction of the machinery involved in translation (Tables S4‐S8, Supporting Information).

Simultaneously, specific factors of stress response and detoxification are induced for example enzymes that contribute to ROS detoxification pathways like catalase and thiol‐peroxidase were upregulated, and in order to maintain cell integrity protein folding mechanisms (chaperons) were increased. De‐repression of bile salt exporting efflux pumps (CmeABC/DEF) by reduced expression of upstream regulatory systems like CmR and CbrR would potentially enable reduction of local bile acid concentrations.

Furthermore, the *C. jejuni* energy metabolism is streamlined, i.e., components of the more energy‐efficient aerobic respiration pathway were increased after exposure to DCA and CDCA, while the expression level of factors of the energy‐inefficient anaerobic branches of the ETC were lowered.

Thus, rather than a single event, bile salts induce a complex physiological response to which proteins of a variety of functional categories contribute. The present study provides an overview of the complex network of mechanisms that affect cellular physiology in response to bile stress in *C. jejuni*.

## Conflict of Interest

The authors declare no conflict of interest.

## Supporting information

Supporting Information

Supporting Information

Supporting Information

Supporting Information
