# Supplementary material for: Proteome Profiling by Label‐Free Mass Spectrometry Reveals Differentiated Response of Campylobacter jejuni 81–176 to Sublethal Concentrations of Bile Acids
Source: Proteomics Clin Appl. 2018 Oct 11;13(3):1800083. doi: 10.1002/prca.201800083 (PMC6585709; doi:10.1002/prca.201800083)
Supplement: Supplementary file 1 — Supporting Information [file PRCA-13-na-s001.docx]

**Supplementary Figures**

**Supplementary Figure 1: Growth curves of C. jejuni 81-176 in pure CDB and CDB supplemented with bile acids at a concentration of ½ IC_50._**

**
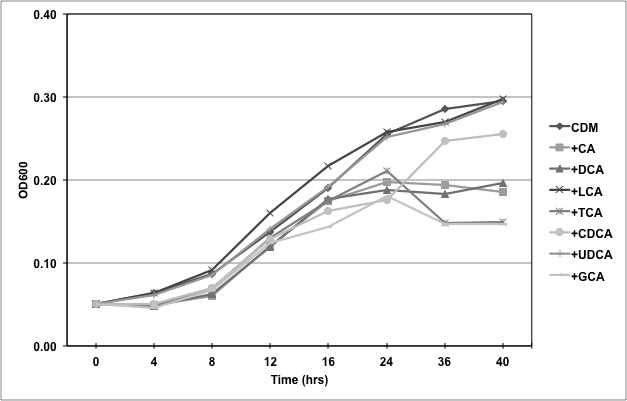
**

The plotted values represent an average of three measurements at different occasions.

**Supplementary Tables**

**Supplementary Table 1**: Measured IC_50_ values of bile acids used in this study and calculated half IC_50_ concentrations used for proteome analysis

|  | **Bile acid** |  | **IC_50_ ± SD**  **%m/m (mM)** |  | **Half IC_50_**  **%m/m (mM)** |  |
| --- | --- | --- | --- | --- | --- | --- |
|  | **CA** |  | 0.15% ± 0.05 (3.48) |  | 0.075% (1.74) |  |
|  | **CDCA** |  | 0.10% ± 0.10 (2.41) |  | 0.05% (1.21) |  |
|  | **TCA** |  | 0.96% ± 0.23 (18.04) |  | 0.485% (9.02) |  |
|  | **GCA** |  | 0.74% ± 0.19 (15.18) |  | 0.370% (7.59) |  |
|  | **DCA** |  | 0.06% ± 0.04 (1.45) |  | 0.030% (0.72) |  |
|  | **LCA** |  | 1.00% ± 0.25 (26.56) |  | 0.50% (13.28) |  |
|  | **UDCA** |  | 0.970% ± 0.22 (24.71) |  | 0.485% (12.35) |  |

**Supplementary Table 2**: *Campylobacter jejuni* 81-176 proteins identified by LC/MS/MS at 1% FDR. The table lists the ProteinPilot Unused Score (‘Score’), Sequence Coverage (SC) total and substantiated by ≥95% confident peptides, and the number of distinct ≥95% confident peptide sequences per protein.

**Supplementary Table 3**: *Campylobacter jejuni* 81-176 proteins quantified by SWATH-MS analysis at 1% FDR. The table lists the summed Protein Peak Areas per protein and replicate.

**Supplementary Table 4: Nucleotide biosynthesis**

Nucleotide biosynthesis is essential for bacteria to maintain their cell integrity and to proliferate. In our proteome analysis for *C. jejuni* we could show that DCA, CDCA, and/or GCA down-regulated various key enzymes of nucleotide biosynthesis including adenine phosphoribosyltransferase Apt, dCTP deaminase Dcd, nucleotide pyrophosphatase Maf ^[1]^, phosphoribosyl-formylglycinamidine cyclo-ligase PurM, phosphoribosyl-formylglycinamidine synthase subunit PurS, dihydro-orotate dehydrogenase PyrD, orotate phosphoribosyl-transferase PyrE, and CTP synthase PyrG. This leads to the conclusion that the suppression of nucleotide biosynthesis is an adaptation to reduced replication rates in the presence of bile acids.

| Uniprot  accession |  | gene |  | protein function | CDCA | DCA | GCA | CA | TCA | UDCA | LCA |
| --- | --- | --- | --- | --- | --- | --- | --- | --- | --- | --- | --- |
| A1VZR0 |  | *apt* |  | adenine phosphoribosyltransferase |  |  |  |  |  |  |  |
| A0A0H3PA20 |  | *dcd* |  | dCTP deaminase |  |  |  |  |  |  |  |
| A1VYL9 |  | *maf* |  | Maf-like protein |  |  |  |  |  |  |  |
| Q0Q7I1 |  | *purM* |  | phosphoribosylformylglycinamidine cyclo-ligase |  |  |  |  |  |  |  |
| A0A0H3PBK5 |  | *purS* |  | phosphoribosylformylglycinamidine synthase |  |  |  |  |  |  |  |
| A0A0H3PA89 |  | *pyrD* |  | dihydroorotate dehydrogenase |  |  |  |  |  |  |  |
| A1VXV5 |  | *pyrE* |  | orotate phosphoribosyltransferase |  |  |  |  |  |  |  |
| A1VXA6 |  | *pyrG* |  | CTP synthase |  |  |  |  |  |  |  |

Color code for all tables: blue – bile acids with log2FC >0.585 (significantly increased expression); orange – bile acids with log2FC <-0.585 (significantly reduced expression); yellow – bile acids with log2FC >-0.585 x <0.585 (not significantly altered in expression), grey – proteins not detected in experimental setting

**Supplementary Table 5: DNA repair factors upregulated by bile acids**

DOC-induced ROS are known to cause double strand breaks ^[2]^, therefore *C. jejuni* should repair the DNA damage to survive. Gourley and coworkers demonstrated that the AddAB recombination complex plays a significant role in the resistance to DOC and is involved in ROS-induced DNA repair ^[3]^. Furthermore they have shown that the AddAB system is necessary for efficient colonization of the chicken intestine by *C. jejuni* ^[3]^.

However, due to our proteomic data enzymes involved in DNA repair including AddA, AddB, Nth, RadA, and LigA are down-expressed under bile acid exposition. As listed in Table 7 AddA has changed significantly in their expression under CDCA exposure and AddB under DCA, CDCA, and GCA exposure.

| Uniprot  accession |  | gene |  | protein function | CDCA | DCA | GCA | CA | TCA | UDCA | LCA |
| --- | --- | --- | --- | --- | --- | --- | --- | --- | --- | --- | --- |
| A0A0H3P9V7 |  | *cjj81176_1101* |  | DNA repair, endonuclease |  |  |  |  |  |  |  |
| A0A0H3PB11 |  | *addA* |  | ATP-dependent DNA helicase AddA, UvrD/REP family |  |  |  |  |  |  |  |
| A0A0H3PDK8 |  | *addB* |  | AddAB recombination complex, helicase AddB |  |  |  |  |  |  |  |
| A0A0H3PEB4 |  | *nth* |  | DNA repair, endonuclease |  |  |  |  |  |  |  |
| A0A0H3PAG5 |  | *radA* |  | DNA repair protein |  |  |  |  |  |  |  |
| A0A0H3PJI4 |  | *recN* |  | DNA repair protein |  |  |  |  |  |  |  |
| A1VYU6 |  | *ligA* |  | DNA ligase |  |  |  |  |  |  |  |

**Supplementary Table 6: Protein synthesis and protein folding factors modulated by bile acids**

A major component of cell damage, particularly by DCA and CDCA, is the down-regulation of translation and thus down-regulation of the replacement of damaged proteins that should maintain cell integrity under stress conditions. Our experimental data indicated a decreased expression of several aminoacyl-tRNA synthetases and of *Gln*-tRNA-amidotransferase (subunits A, B, C). Translation initiation factor IF-3, and other proteins involved in the translation process (Table 8).

In contrast some ribosomal proteins, namely S8, S20, L1, L23, L33, and RbfA were significantly increased in their expression under bile acid exposure, while ribosomal proteins S3, L9, and L16 were significantly down-regulated as a result of bile acid exposition (Table 8). As a consequence of the toxic influence of bile acids, total protein synthesis was significantly reduced.

According to our analysis, most of the detectable chaperones (DnaJ-1, GroEL, DsbA, DsbB, and DsbD) were up-expressed under bile acid exposure. Accordingly it seems that the bacterial organism attempts to compensate for the decreased protein synthesis by increasing protein repair.

| Uniprot  accession |  | gene |  | protein function | CDCA | DCA | GCA | CA | TCA | UDCA | LCA |
| --- | --- | --- | --- | --- | --- | --- | --- | --- | --- | --- | --- |
| A1VYL8 |  | *alaS* |  | alanyl-tRNA synthetase |  |  |  |  |  |  |  |
| A1W0F9 |  | *argRS* |  | arginyl-tRNA synthetase |  |  |  |  |  |  |  |
| A1VZ00 |  | *aspS* |  | aspartyl-tRNA synthetase |  |  |  |  |  |  |  |
| A0A0H3PID1 |  | *glyS* |  | glycyl-tRNA synthetase |  |  |  |  |  |  |  |
| A1W0S2 |  | *gluRS 2* |  | glutamyl-tRNA synthetase |  |  |  |  |  |  |  |
| A1VZB3 |  | *hisRS* |  | histidyl-tRNA synthetase |  |  |  |  |  |  |  |
| A0A0H3PAI4 |  | *ileRS* |  | isoleucyl-tRNA synthetase |  |  |  |  |  |  |  |
| A1W078 |  | *leuRS* |  | leucyl-tRNA synthetase |  |  |  |  |  |  |  |
| A1VYC1 |  | *lysRS* |  | lysyl-tRNA synthetase |  |  |  |  |  |  |  |
| A0A0H3P9K7 |  | *metRS* |  | methionyl-tRNA synthetase |  |  |  |  |  |  |  |
| A1VZN1 |  | *pheS* |  | phenylalanyl-tRNA synthetase |  |  |  |  |  |  |  |
| A0A0H3PHR2 |  | *pheT* |  | phenylalanyl-tRNA synthetase |  |  |  |  |  |  |  |
| A1VYA9 |  | *serRS* |  | seryl-tRNA synthetase |  |  |  |  |  |  |  |
| A1VXT5 |  | *thrRS* |  | threonyl-tRNA synthetase |  |  |  |  |  |  |  |
| A0A0H3PB64 |  | *trpRS* |  | tryptophanyl-tRNA synthetase |  |  |  |  |  |  |  |
| A0A0H3PDU5 |  | *tyrRS* |  | tyrosyl-tRNA synthetase |  |  |  |  |  |  |  |
| A0A0H3PHD8 |  | *valRS* |  | valyl-tRNA synthetase |  |  |  |  |  |  |  |
| A1VYQ2 |  | *proS* |  | prolyl-tRNA synthetase |  |  |  |  |  |  |  |
| A1W165 |  | *truD* |  | tRNA pseudouridine synthase |  |  |  |  |  |  |  |
| A1W048 |  | *gatA* |  | tRNA(Gln) amidotransferase |  |  |  |  |  |  |  |
| A1W0I1 |  | *gatB* |  | tRNA(Asn/Gln) amidotransferase |  |  |  |  |  |  |  |
| A1VYB8 |  | *gatC* |  | tRNA(Asn/Gln) amidotransferase |  |  |  |  |  |  |  |
| A1VZZ8 |  | *tgt* |  | tRNA-guanine transglycosylase |  |  |  |  |  |  |  |
| A1VZ24 |  | *arG* |  | argininosuccinate synthase |  |  |  |  |  |  |  |
| A1VY95 |  | *yajQ* |  | UPF0234 protein YajQ |  |  |  |  |  |  |  |
| A1VXT6 |  | *infC* |  | translation initiation factor IF-3 |  |  |  |  |  |  |  |
| A0A0H3PCJ0 |  | *cjj81176_0101* |  | site-determining protein |  |  |  |  |  |  |  |
| A0A0H3PBY2 |  | *cjj81176_0318* |  | ThiF family protein |  |  |  |  |  |  |  |
| A0A0H3PBL2 |  | def |  | polypeptide deformylase |  |  |  |  |  |  |  |
| A0A0H3P9B1 |  | *yajC* |  | preprotein translocase |  |  |  |  |  |  |  |
| A1VYU1 |  | *rppH* |  | RNA pyrophosphohydrolase |  |  |  |  |  |  |  |
| A1W1L3 |  | *rpsT* |  | 30S ribosomal protein S20 |  |  |  |  |  |  |  |
| A1W1U6 |  | *rpsH* |  | 30S ribosomal protein S8 |  |  |  |  |  |  |  |
| A1VYJ1 |  | *rplA* |  | 50S ribosomal protein L1 |  |  |  |  |  |  |  |
| A1VZ23 |  | *rplI* |  | 50S ribosomal protein L9 |  |  |  |  |  |  |  |
| A1W1V3 |  | *rplP* |  | 50S ribosomal protein L16 |  |  |  |  |  |  |  |
| A1W1V8 |  | *rplW* |  | 50S ribosomal protein L23 |  |  |  |  |  |  |  |
| A1VXH8 |  | *rpmA* |  | 50S ribosomal protein L27 |  |  |  |  |  |  |  |
| A1VYI7 |  | *rpmG* |  | 50S ribosomal protein L33 |  |  |  |  |  |  |  |
| A1W1V4 |  | *rpsC* |  | 30S ribosomal protein S3 |  |  |  |  |  |  |  |
| A0A0H3PDX5 |  | *rnc* |  | ribonuclease 3 |  |  |  |  |  |  |  |
| A0A0H3PBB3 |  | *rbfA* |  | ribosome-binding factor A |  |  |  |  |  |  |  |
| A0A0H3PB76 |  | *dnaJ-1* |  | protein folding, chaperone |  |  |  |  |  |  |  |
| Q0Q7K7 |  | *dnaK* |  | chaperone protein |  |  |  |  |  |  |  |
| A1W0K4 |  | *groEL* |  | chaperonin |  |  |  |  |  |  |  |
| A0A0H3PA52 |  | *htrA* |  | chaperone, Protease |  |  |  |  |  |  |  |
| A1VYN0 |  | *htpG* |  | protein folding, chaperone |  |  |  |  |  |  |  |
| A0A0H3PA35 |  | *dsbA* |  | protein folding, Thiol:disulfide interchange protein |  |  |  |  |  |  |  |
| A0A0H3PCI0 |  | *dsbB* |  | protein folding, disulfide bond formation protein |  |  |  |  |  |  |  |
| A0A0H3PBJ5 |  | *dsbD* |  | protein folding, Thiol:disulfide interchange protein |  |  |  |  |  |  |  |

**Supplementary Table 7: Lipid and carbohydrate biosynthesis under bile acid stress**

It is well known that bile acids damage the cell wall of enteric bacteria, like *C. jejuni* ^[4]^. As the bacterial cell is not only composed of proteins but also lipids and carbohydrates, increased lipid and carbohydrate biosynthesis was assumed to be another aspect to maintain cell membrane and cell wall integrity under bile acid stress ^[5]^.

According to our proteomic analysis in the context of a general diminished protein synthesis by the toxic influence of bile acids, especially by DCA and CDCA, most lipid biosynthesis proteins (IpxB, IpxD, AcpP, GpsA), fatty acids biosynthesis proteins (FabF, AcpP) and carbohydrate biosynthesis proteins (RpIB, GmhA-1, Pgi, Fbp, CJJ81176_1247, GmhA-2) were significantly down-expressed (Table 9).

In contrast, TCA significantly increased expression of the inositol-1-monophosphatase CysQ. This protein guides the arrangement of proteins into the cell membrane, periplasmic membrane and cell wall ^[6]^.

| Uniprot  accession |  | gene |  | protein function | CDCA | DCA | GCA | CA | TCA | UDCA | LCA |
| --- | --- | --- | --- | --- | --- | --- | --- | --- | --- | --- | --- |
| A0A0H3PEG0 |  | *IpxB* |  | lipid-A-disaccharide synthetase |  |  |  |  |  |  |  |
| A0A0H3PAD5 |  | *IpxD* |  | UDP-3-O-acylglucosamine N-acyltransferase |  |  |  |  |  |  |  |
| A1VYF9 |  | *acpP* |  | acyl carrier protein |  |  |  |  |  |  |  |
| A1W0I0 |  | *gpsA* |  | glycerol-3-phosphate dehydrogenase |  |  |  |  |  |  |  |
| A0A0H3PF03 |  | *fabF* |  | 3-oxoacyl synthase 2 |  |  |  |  |  |  |  |
| A0A0H3P9T0 |  | *gmhA-1* |  | phosphoheptose isomerase |  |  |  |  |  |  |  |
| Q29VW1 |  | *gmhA-2* |  | phosphoheptose isomerase |  |  |  |  |  |  |  |
| A0A0H3PAP2 |  | *pgi* |  | Glc-6-phosphate isomerase |  |  |  |  |  |  |  |
| A1VZI4 |  | *fbp* |  | fructose-1,6-bisphosphatase |  |  |  |  |  |  |  |
| A0A0H3PI47 |  | *cjj81176_1247* |  | HAD hydrolase IA phosphosugar phosphatase |  |  |  |  |  |  |  |
| A0A0H3P9S5 |  | *cysQ* |  | inositol-1-monophosphatase |  |  |  |  |  |  |  |

**Supplementary Table 8: Amino acid uptake, synthesis and utilization** **under bile acid influence**

*C. jejuni* utilizes mainly scavenging amino acids and keto acids from the host or from its intestinal microbial flora as carbon sources in a sequential pattern ^[7]^. Specific *C. jejuni* clades expressing a periplasmic γ-glutamyltranspeptidase (Ggt) and a periplasmic asparaginase (AnsB) show an extended amino acid metabolism ^[8,9]^.

The global picture of our mass spectrometric analysis of the amino acid metabolism in *C. jejuni* under bile acid influence is very heterogeneous.

Our proteomic analysis (Table 10) showed that carbon starvation protein A (CstA) that is known to be a peptide uptake system ^[10]^, serine transporter SdaC, sodium:alanine symporter SAS, glutamate synthase GltB & GltD, and glutamine synthetase GlnA were up-expressed under DCA and CDCA exposure.

In contrast the ABC transporter Peb1A & Peb1C, sodium/proline permease PutP, histidine biosynthesis bifunctional protein HisIE, apspartate aminotransferase AspC, and prephenate dehydrogenase TyrA showed lowered expression under bile acid influence.

Although the majority of amino acids were taken up from the environment, a proportion is also synthesized by the bacterial cell. Thus, our study revealed that during growth in DCA, GCA and CDCA, *C. jejuni* 81-176 simultaneously up-regulated proteins for the biosynthesis of glutamate and glutamine, but down-regulated histidine, aspartate, and tyrosine synthesis.

One enzyme of the extended amino acid metabolism, Ggt, in *C. jejuni* 81-176 was up-expressed in consequence of CA, LCA, and TCA exposition, while AnsB was down-expressed by CA, DCA, CDCA, and GCA.

According to Ruiz and colleagues, this significant upward adjustment in requirements of energy assists bacteria growing in the presence of bile acids to actively synthesize various response mechanisms against effects of bile acids ^[11]^.

| Uniprot  accession |  | gene |  | protein function | CDCA | DCA | GCA | CA | TCA | UDCA | LCA |
| --- | --- | --- | --- | --- | --- | --- | --- | --- | --- | --- | --- |
| A0A0H3PCE2 |  | *cstA* |  | carbon starvation protein A |  |  |  |  |  |  |  |
| A1VZQ4 |  | *peb1A* |  | amino acid transport |  |  |  |  |  |  |  |
| A1VZQ5 |  | *peb1C* |  | amino acid transport |  |  |  |  |  |  |  |
| A0A0H3PDM3 |  | *sdaC* |  | serine transporter |  |  |  |  |  |  |  |
| A0A0H3PA18 |  | *sas* |  | sodium:alanine symporter |  |  |  |  |  |  |  |
| A0A0H3PA17 |  | *putP* |  | sodium/proline permease |  |  |  |  |  |  |  |
| A0A0H3PAJ4 |  | *hisIE* |  | histidine biosynthesis bifunctional protein |  |  |  |  |  |  |  |
| Q1HG74 |  | *gltB* |  | glutamate synthase |  |  |  |  |  |  |  |
| Q1HG72 |  | *gltD* |  | glutamate synthase |  |  |  |  |  |  |  |
| A0A0H3PHD6 |  | *glnA* |  | glutamine synthetase |  |  |  |  |  |  |  |
| A0A0H3PGM1 |  | *aspA* |  | aspartate ammonia-lyase |  |  |  |  |  |  |  |
| A0A0H3P9M4 |  | *aspC* |  | aspartate-aminotransferase |  |  |  |  |  |  |  |
| A0A0H3P9R4 |  | *sdaA* |  | L-serine ammonia-lyase |  |  |  |  |  |  |  |
| A0A0H3PBI3 |  | *putA* |  | proline dehydrogenase |  |  |  |  |  |  |  |
| A0A0H3PAH1 |  | *tyrA* |  | prephenate dehydrogenase |  |  |  |  |  |  |  |
| A0A0H3PA64 |  | *ggt* |  | gamma-glutamyl-transferase |  |  |  |  |  |  |  |
| A0A0H3PCK6 |  | *ansB* |  | L-asparaginase |  |  |  |  |  |  |  |

**References**

[1] A. Tchigvintsev, D. Tchigvintsev, R. Flick, A. Popovic, A. Dong, X. Xu, G. Brown, W. Lu, H. Wu, H. Cui, L. Dombrowski, J. C. Joo, N. Beloglazova, J. Min, A. Savchenko, A. A. Caudy, J. D. Rabinowitz, A. G. Murzin, A. F. Yakunin, *Chem. Biol.* **2013**, *20*, 1386.

[2] N. M. Negretti, C. R. Gourley, G. Clair, J. N. Adkins, M. E. Konkel, *Sci. Rep.* **2017**, *7*, 15455.

[3] C. R. Gourley, N. M. Negretti, M. E. Konkel, *Sci. Rep.* **2017**, *7*, 14777.

[4] M. Begley, C. G. M. Gahan, C. Hill, *FEMS Microbiol. Rev.* **2005**, *29*, 625.

[5] M. E. Merritt, J. R. Donaldson, *J. Med. Microbiol.* **2009**, *58*, 1533.

[6] G. Di Paolo, P. De Camilli, *Nature* **2006**, *443*, 651.

[7] M. Stahl, J. Butcher, A. Stintzi, *Front. Cell. Infect. Microbiol.* **2012**, *2*, 5.

[8] D. Hofreuter, V. Novik, J. E. Galan, *Cell Host Microbe* **2008**, *4*, 425.

[9] A. E. Zautner, S. Herrmann, J. Corso, A. M. Tareen, T. Alter, U. Gross, *Appl Env. Microbiol* **2011**, *77*, 2359.

[10] J. J. Rasmussen, C. S. Vegge, H. Frøkiær, R. M. Howlett, K. A. Krogfelt, D. J. Kelly, H. Ingmer, *J. Med. Microbiol.* **2013**, *62*, 1135.

[11] L. Ruiz, A. Margolles, B. Sánchez, *Front. Microbiol.* **2013**, *4*, 396.
