# Supplementary figures and images for: Proteome Profiling by Label‐Free Mass Spectrometry Reveals Differentiated Response of Campylobacter jejuni 81–176 to Sublethal Concentrations of Bile Acids
Source: Proteomics Clin Appl. 2018 Oct 11;13(3):1800083. doi: 10.1002/prca.201800083 (PMC6585709; doi:10.1002/prca.201800083)

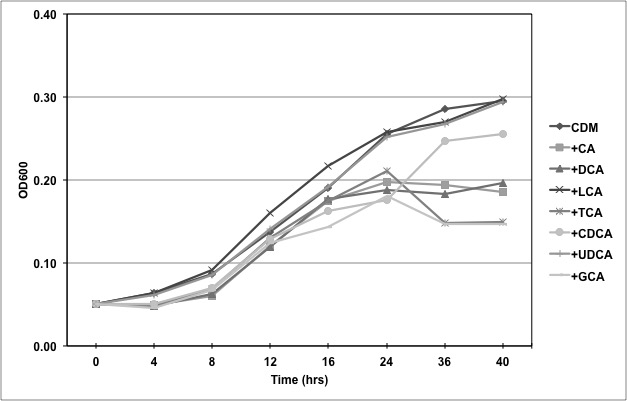

Supplement: Supplementary file 2 — Supporting Information [file PRCA-13-na-s002.jpg]
